# Social Service in the Temperance Reformation

**Published:** 1908-01

**Authors:** Cora Frances Stoddard

**Affiliations:** Corresponding Secretary of the Scientific Temperance Federation, Boston


### Social Service in the Temperance Reformation.

BY CORA FRANCES STODDARD,
Corresponding Secretary of the Scientific Temperance Federation, Boston.

A hasty review of the contents of the leading American periodicals of the last three months reveals more than fifty serious articles on questions of social relations and responsibilities. They touch almost every phase of human existence, but all approach it from the standpoint of the essential oneness of mankind.

It is no longer sufficient that a man be physically, mentally and morally sound for his own sake; or that he have his political and material rights for his personal comfort and advancement. The progress of one is so interlaced with that of all, that work for social uplift begun in compassion becomes a measure of self-protection and for the advancement of all society. The giving of life for one's fellow man is the finding of life for humanity. It is this which is the essential principle in all social movements whether they have as their object the removal or the amelioration of evil conditions.

### THE BREADTH OF SOCIAL SERVICE.

The field of social effort is as wide as the sorrows and failings of all humanity. "Social workers" are not in a select class by themselves. There are specialists in this as in any other field of activity, but the man who insists on renting only healthful tenements, who guards the health and safety of his employes; the teacher who trains her pupils to noble ideals, healthful habits and intelligent abstinence from narcotics; the mother who rears her boys and girls to a high sense of the purity and sanctity of the home, are as truly engaged in social service as the man who builds a public library, endows a people's palace or hospital, or lives in a social settlement in the slums.

The question is not, therefore, whether any special work for one's fellow man is a part of social endeavor. It is rather, what is the relation of that work to the whole movement of social uplift? The settlement worker may not say to the temperance worker, "I have no need of thee." The temperance worker may not say to the advocate of tenement house reform or of domestic science, "I have no need of thee." All are necessary to one another. Each sees with special clearness a side of social truth. Hence each may learn of the other.

### THE RIGHT PERSPECTIVE.

Recognition of this fact in relation to what is popularly termed "temperance work" is of peculiar importance, both from the standpoint of those engaged in it and from that of other social workers. Too long there has been a tendency to isolate this, and to regard it as something distinct from other social efforts, its advocacy a mere indication of "crankiness" and of a desire to press personal prejudices upon the world at large. This is both unreasonable and contrary to the spirit of social science, and it is quite time that this work should be given as a matter of course its proper rightfully dignified place as a branch of social endeavor.

What, then, is the place of temperance work in social uplift?

No one claims, of course, that the elimination of alcohol and other narcotics would solve all social problems. But the relation is far more fundamental than is often recognized. Many a social worker toiling heroically at his relief or preventive measures might well face squarely the facts of the violation of social laws by drink and other narcotics and their bearings upon his special problem, for the use of these substances has an intimate relation to three purposes with which society concerns itself—self-preservation, happiness and efficiency.

SOCIAL WRONGS AGAINST THE STRENGTH OF YOUTH.

We have learned by long, painful experience that the motive powers of civilization are those elements that make the perfect man. The race, if it is to be preserved from physical degeneracy, can not be propagated from the poorest conditions, and high moral ideals are the "spiritual police" which safeguard civilization in its perilous progress. Whatever tends to lower these ideals or to impair physical perfection must therefore be combated by society in self-protection.

A delegate from the Christian Democrats said recently at a meeting in Belgium for founding an organization to work against alcohol, "Society has the right to protect itself against the excesses of certain of its members and to protect these unfortunates against themselves. The struggle against alcohol is a defensive movement and a work for solidarity." A British Royal Commission has pronounced the use of alcohol one of the most potent causes of physical deterioration. Careful experiments and observations of the scientists are proving that the descendants of alcohol users are less likely to live, a greater proportion are weak physically and mentally, and not only threaten the integrity of the race, but are themselves liable to become dependent upon State or private charity.

The report of the Massachusetts State Board of Insanity for 1906 showed that of the admissions to public insane hospitals during the year, alcohol was a factor in 21.66 per cent. If the same percentage holds for other inmates of the asylums, in the one State of Massachusetts, there are 2190 insane persons whose condition is due, at least in part, to alcohol. Their support cost the State in one year $660,000. The superintendent of an Illinois insane hospital recently stated that there are nearly 2000 epileptics in the public institutions of that State "who could, if they knew enough, point their fingers at one or both of their parents and say, 'You are responsible for my misery through alcohol which made you its slave.' "

But what of those spiritual ideals which are the guiding star of civilization? Again, science tells us that alcohol acts most quickly upon the higher mental faculties, impairing judgment, will, reason and those finer mental and moral attributes which are the distinguishing mark of the divine element in man, qualities upon which the success of the teacher and all other elevating forces of society must build if the present or the future of the race is to be conserved. No other force does more to lower, or to keep on a low plane, ideals in home life which many an earnest social settlement dweller is trying to raise. "The school, the church, the college, the newspaper, can never effectually give culture to children whose home life is vulgar and wicked."

### HAPPINESS-MAKERS AND HAPPINESS-BREAKERS.

A second end of social endeavor is to secure a higher degree of happiness. This is the meaning of the homes for destitute children, of the innumerable charitable aid societies, of rescue homes, of settlement activities, and of almost innumerable other organized forms of social helpfulness.

The expenditures for these purposes in money alone are as stupendous as the devotion of time and energy of some of the choicest of lives is inspiring. Though the financial expense is one of the least of the evils, it furnishes a concrete illustration of the waste which alcohol causes. Of the nearly 250 private charitable organizations in Boston, 30 alone spent, in 1906, nearly $800,00 in varied efforts to relieve distress. Using the most conservative estimates, not far from $300,000 of this money was expended in alleviating the results of the use of alcohol, and of this amount about $150,000 was used alone in caring for children made destitute or abused by drink.

### THE CRY OF THE CHILDREN.

There is indeed "a bitter cry of the children." The Massachusetts Society for the Prevention of Cruelty to Children has reported that 65 per cent of its cases were the innocent victims of drink. Jacob Riis tells us in *The Children of the Poor* that seven out of every ten street boys in New York, who have any homes, left them because of drunkenness in the home.

Not only the pitiably destitute, but thousands of children too early turned into the machine of merciless labor, are the helpless and innocent victims of alcohol which has made them the bond-servants of poverty in the home. "Poverty and child labor are yoke fellows," says Mr. Riis, and careful investigations have shown that not less than 25 per cent of all poverty which seeks public relief is due to the use of alcoholic beverages. To this extent the child labor problem and the alcohol question are one, but they are one in the sense that the tree and the root are one. Cut off the root of alcohol and many a flourishing branch of the deadly child labor tree will wither and die.

In 1906, the State of Massachusetts expended about $500,000 in caring for the pauperism in which alcohol was a factor; $450,000 expense for prisoners whose offenses were partly due to drink. It cost the public treasury of Boston about $1,500,000 for the crime in which alcohol had a part, and $480,000 for crime in which alcohol was the sole cause.

The financial waste is sad enough and bad enough, but what shall be said of the unspeakable misery and waste of human life and powers, of the demand it makes upon the sympathies, of the difficulties in maintaining and increasing moral sense in society? To alleviate misery is well. To enable victims to escape from it by their own purposeful efforts is still better; but to leave untouched the great cause of a vast amount of this misery is like "picking the jewel from the mud puddle," and leaving the puddle in the way of the next traveler.

SOCIAL EFFICIENCY.

Life is not merely existence. It is measured as well in terms of accomplishment. Every man owes to his day all of which he is capable by natural endowment and by training. He must ever be at his best to meet the demands made upon him. A few months or even days, requiring quick judgment, prompt decision, alertness to opportunity, may be the turning point in the current not only of his own life, but not infrequently of his entire generation. It is, therefore, the right of society to expect the utmost possible efficiency of its members. For this we establish kindergartens, schools, technical institutions, gymnasiums, public lectures and concerts, hospitals and dispensaries for repairing weakness with as little loss and delay as possible, and a thousand and one other institutions for developing, training and conserving all the powers with which man is endowed.

But when all these have done their work at immense public and private cost, society may again be checkmated by alcohol and its narcotic friends. Muscle is paralyzed; nerve control involving precision and delicacy of touch, judgment and power to think clearly, quickly and intelligently, is impaired. The children of alcoholic parents appear to be handicapped from the start with tendencies to dullness or backwardness. Bad air, lack of food or of proper clothing may be ascribed as causes of inefficiency, but in a vast number of cases alcohol will be found the ultimate cause, a subtle mischief maker. As a result, instead of a happy, intelligent, efficient social order, our social system is weighted with a great burden of incompetents, many of whom by reason of alcoholic heredity are not responsible for their condition, but who, nevertheless, are so many clogs in the wheels of progress, and who will hand on to the next generation a still greater burden of inefficiency.

Much might be said, too, of the operations of kindred evils engendered by other narcotics. Consequences vary with the substance used, but the net result is similar in kind if not always in a degree,—a lowering of ideals, impairment of physical anl mental powers, a blunting of the sense of social responsibility. The cigarette habit among boys emulating the example of their elders is poisoning the very fountain head of our future civilization. Mr. W. L. Bodine, Superintendent of Compulsory Education in Chicago public schools, in a recent address stated that of 1015 boys whom he had been obliged to send to the Parental School, about 800 were cigarette smokers. Of the entire number only 145 were up in their school grades. These 800 boys might be many times multiplied all over our land. What can be expected of these boys in efficiency in life's work or of their sons and daughters in the next generation?

This waste of physical and mental powers, of moral ideals, of happiness and efficiency constitutes a fact too serious to be ignored by anyone having the smallest sense of social responsibility. The alcohol and narcotic questions, so far from being separate problems, become the problems of all, as they are an opportunity for all. "The temperance movement," Prof. Richard T. Ely has said, "is a deep, wide movement of social reform which centers in temperance, but from that center it spreads out in ever more and more inclusive circles until it touches the entire life of society." Alcoholism and narcotism are both cause and effect of social ills. Just so far as these elements can be eliminated, the whole social problem will be simplified.

### GETTING AT THE ROOT OF THE PROBLEM.

The necessary methods for dealing with this all-pervading social question are varied, but it is perfectly evident that the great stronghold of permanent success lies in education. One hundred and twenty years ago, Casper Von Voght, the leader of modern scientific benevolence, said, "The most effective means of preventing misery is the better education of the children," and the great leader of modern scientific temperance instruction, Mary H. Hunt, once said, "Seek ye first the temperance education of the children, and all other temperance blessings shall be added unto you." In either case, *prevention* is the keynote. Not much can be done with the victims of alcohol and other narcotics today. Practically everything can be done eventually through the proper training of the children and youth. The place of temperance education in the work for social uplift is therefore natural and fundamental.

Because of its inter-relations, such education must be many-sided. Knowing, feeling, doing are the educational trinity. Complete temperance education, therefore, must reach the reason, the conscience and the will. The child must be equipped with knowledge of the facts as to the character of alcoholic drinks and of other narcotics and the dangers in their use. These truths should be taught dispassionately, truthfully, convincingly by the teacher who knows her subject thoroughly. Secondly, the pupils must be inspired with high ideals for life and character, and with the majesty and beauty of self-control which subordinates the baser, physical appetites to the pleasures of intellectual and spiritual powers. And, lastly, temperance education must lead the child to voluntary choice of habits of healthful living. Use rationally the first two elements of training and the last will follow naturally and almost easily. But no one of these three may reasonably be omitted.

### "TILL THE LUMP BE LEAVEN."

It may be readily admitted that the task presented is no easy one. It demands the teachers' best powers, greatest tact, adequate knowledge, and infinite patience, but, "difficulties have been yielding to enthusiasm ever since the world began." *And it pays.* Count it no small thing in the record of the year to have set even one child's feet permanently in the path of intelligent self-reverence and control. Society is made up of these little units. All growth in the physical organism is but the result of the working of tiny cells. So, in the social organism, the unit of growth is the individual life. The 300,000 teachers of the United States, if each saves but one child each year, are planting the leaven of social progress in the midst of the common life of every 300 inhabitants of the whole nation.

COMRADES OF THE GREATER FAITH.

Such are the opportunities and the dignity of temperance work. It is not the temperance "reform," merely seeking to reclaim the lost, or to propagate the prejudices of a few enthusiasts. It is rather the temperance "reformation" which is thoroughly reconstructive and preventive. It is governed by the laws of social relations. It is animated by the common impulse of all social effort, viz., the redemption of man to his divine self. This work, someone has said, "can never be separated, in theory or in practice from that general progress of humanity by which the will of God is made to prevail all around the orbit of human life and in all the sons of men." The very vastness of this problem, therefore, gives it a commanding dignity in its place in social reformation. It constitutes a task which from its inherent difficulties should compel sympathy, and stir to action those native human forces which respond with eagerness to a call summoning to service the best thought and powers of twentieth century manhood and womanhood.—*School Physiology Journal.*

